# Bottom Contact 100 nm Channel‐Length α‐In_2_Se_3_ In‐Plane Ferroelectric Memory

**DOI:** 10.1002/advs.202303032

**Published:** 2023-08-11

**Authors:** Shurong Miao, Ryosuke Nitta, Seiichiro Izawa, Yutaka Majima

**Affiliations:** ^1^ Laboratory for Materials and Structures Institute of Innovative Research Tokyo Institute of Technology Yokohama Kanagawa 226‐8503 Japan; ^2^ Joining and Welding Research Institute Osaka University Ibaraki Osaka 567‐0047 Japan

**Keywords:** bottom contact, in‐plane ferroelectric memory, nanogap electrodes, nano‐channel memory, α‐In_2_Se_3_

## Abstract

Owing to the emerging trend of non‐volatile memory and data‐centric computing, the demand for more functional materials and efficient device architecture at the nanoscale is becoming stringent. To date, 2D ferroelectrics are cultivated as channel materials in field‐effect transistors for their retentive and switchable dipoles and flexibility to be compacted into diverse structures and integration for intensive production. This study demonstrates the in‐plane (IP) ferroelectric memory effect of a 100 nm channel‐length 2D ferroelectric semiconductor α‐In_2_Se_3_ stamped onto nanogap electrodes on Si/SiO_2_ under a lateral electric field. As α‐In_2_Se_3_ forms the bottom contact of the nanogap electrodes, a large memory window of 13 V at drain voltage between ±6.5 V and the on/off ratio reaching 10^3^ can be explained by controlled IP polarization. Furthermore, the memory effect is modulated by the bottom gate voltage of the Si substrate due to the intercorrelation between IP and out‐of‐plane (OOP) polarization. The non‐volatile memory characteristics including stable retention lasting 17 h, and endurance over 1200 cycles suggest a wide range of memory applications utilizing the lateral bottom contact structure.

## Graphical abstract

In‐plane (IP) ferroelectric memory effect of a 100 nm channel‐length 2D ferroelectric semiconductor α‐In_2_Se_3_ stamped onto nanogap electrodes on Si/SiO_2_ is demonstrated. The non‐volatile memory characteristics with IP polarization including the on/off ratio reaching 10^3^, stable retention lasting 17 h, and endurance over 1200 cycles suggest a wide range of memory applications utilizing the lateral bottom contact structure.

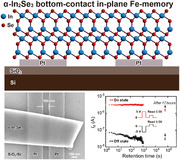

## 1 Introduction

Ferroelectrics are frequently utilized in capacitor‐type ferroelectric random‐access memory (C‐FeRAM),^[^ ^1^, ^2^ ^]^ ferroelectric tunnel junctions (FTJs),^[^ ^3^, ^4^, ^5^ ^]^ and ferroelectric field‐effect transistors (Fe‐FETs)^[^ ^6^, ^7^ ^]^ based on the easy modulation of Schottky barrier height in the interfaces and electrically switched polarization states, which can be interpreted as on/off two current states (“0” and “1”) after electric field sweeping, and are potential for next‐generation non‐volatile memory. The development of C‐FeRAM is hindered due to destructive readout, slow response, and the requirement of large volume which makes massive integration impractical.^[^ ^1^, ^8^ ^]^ For FTJs, the critical thickness of ferroelectrics dominates the electrical performance, where decreasing thickness leads to an increasing depolarization field inside ferroelectrics, decreasing and destabilizing tunnel electron transport, and hence, a relatively low current density, while thicker ferroelectrics cannot allow quantum‐mechanical tunneling.^[^ ^9^ ^]^ Conversely, Fe‐FETs are favorable on account of their low power consumption, fast operation, and non‐destructive readout associated with polarization switching,^[^ ^10^ ^]^ where bulk perovskite ferroelectrics,^[^ ^11^ ^]^ ferroelectric oxides,^[^ ^12^, ^13^ ^]^ and ferroelectric polymers^[^ ^14^ ^]^ are often selected. Nevertheless, ferroelectrics still suffer from thickness scaling. Forming high‐quality thin films with low leakage that ensures complex fabrication remains a crucial problem for commercialization.

After the advent and prevalence of graphene,^[^ ^15^ ^]^ 2D van der Waals (vdW) materials such as MoS_2_,^[^ ^16^ ^]^ h‐BN,^[^ ^17^ ^]^ and CuInP_2_S_6_^[^ ^18^ ^]^ have become increasingly attractive because of the distinguishable ability and the convenience of fabrication of, e.g., transistors, sensors,^[^ ^19^ ^]^ and photodetectors.^[^ ^20^ ^]^ Exhibiting ferroelectricity at the atomic level and an appropriate band gap of 1.43 eV,^[^ ^21^ ^]^ the ferroelectric semiconductor material α‐In_2_Se_3_ is a promising channel material in Fe‐FETs. In contrast to most of the traditional 2D ferroelectrics possessing only one‐direction polarization, the in‐plane (IP) and out‐of‐plane (OOP) polarization originating from symmetry breaking caused by the displacement of Se atom in the center of α‐In_2_Se_3_ nanolayers enables building multistate memristors and neuromorphic computing rectifiers based on a unique interlocking IP and OOP polarization phenomenon.^[^ ^22^ ^]^ It has also been reported that the IP direction has more robust polarization than OOP, about one order of magnitude larger than the OOP dipole,^[^ ^23^ ^]^ which suggests IP polarization has a larger coercive electric field than OOP polarization.

In recent years, α‐In_2_Se_3_ has attracted so much attention in the field of memory. For example, Si et al. proposed a ferroelectric semiconductor field‐effect transistor (FeS‐FET) on α‐In_2_Se_3_.^[^ ^24^ ^]^ Combined with a scaled HfO_2_ gate insulator, the fabricated FeS‐FETs exhibit a high on/off ratio of over 10^8^, a maximum on current of 862 µA µm^−1^. Wang et al. reported 2D α‐In_2_Se_3_ ferroelectric channel transistors with a fast write speed of 40 ns and a series of neural computation performances.^[^ ^25^ ^]^ However, the scope of research is limited by the lack of lateral devices that demonstrate IP polarization‐controlled electrical characteristics. Lateral devices based on IP polarization are not as adequate as vertical ones; in the latter, α‐In_2_Se_3_ is sandwiched by electrodes, and the electrical behaviors are controlled by gate bias.^[^ ^24^, ^26^ ^]^ To improve electrical performance, it is desirable to fully exploit IP polarization for a higher on/off ratio. In addition, the majority of reported devices proposed a channel length greater than or equal to 1 µm.^[^ ^24^, ^26^, ^27^ ^]^ Theoretically, when the channel length is several micrometers long, not all the OOP dipoles inside the channel ferroelectric material can be reoriented and only partial dipoles around the area gapped by the electrodes can be switched.

Scaled‐down Fe‐FETs with nanogap electrodes have merits in achieving a higher on/off ratio, faster response, and faster readout. With a channel length less than sub‐µm, the device is able to modulate polarization under relatively low voltage and show a limited number of threshold voltage (*V*_T_) levels that are related to switching of individual domains,^[^ ^28^ ^]^ enabling novel applications such as random number generators^[^ ^29^ ^]^ and memory cells operated at drive voltage for logic^[^ ^30^ ^]^ and analog^[^ ^25^, ^31^ ^]^ use. When fabricating bottom‐contact Fe‐FETs by 2D material exfoliation, wide electrode width is preferred to improve the overall yield. Nevertheless, achieving nanoscale channel lengths for the nanogap electrodes becomes challenging when simultaneously employing wide electrode widths, mainly due to the substantial ratio between the electrode width and channel length.

To that end, a new‐concept geometry is required to meet the current demands for ultrafast and high‐density non‐volatile memory devices and massively scalable storage. Recently, we established a fabrication technique for Pt‐based nanogap electrodes and demonstrated 2D silicon nanomaterials of a silicane FET with a 130 nm channel length.^[^ ^32^, ^33^ ^]^ Aiming to utilize the IP polarization of vdW material α‐In_2_Se_3_, a nanogap structure that allows the application of lateral electric field can have significant implications for the development of next‐generation non‐volatile memory devices and diverse nanoelectronics.

In this study, we propose a two‐terminal nanogap‐structured bottom contact ferroelectric memory leveraging the IP polarization flipping of α‐In_2_Se_3_. Distinct from previous devices, in our device, α‐In_2_Se_3_ is exfoliated on source and drain electrodes as the bottom contact structure. The IP polarization can be reversed by applying a drain voltage via a channel length down to 100 nm. This α‐In_2_Se_3_ ferroelectric memory exhibits typical resistive switching, a high on/off ratio of over 10^3^, a large memory window of 13 V, good retention for 17 h, and endurance for 1200 cycles, which opens the way for non‐volatile programmed memory.

## 2 Results and Discussion

Piezoresponse force microscopy (PFM), which realizes ferroelectric polarization switching via an external electric field without destroying the sample material, was used to demonstrate ferroelectric characteristics. Nanoflakes were cleaved through mechanical exfoliation of a 2H α‐In_2_Se_3_ crystal and transferred onto both conductive Pt and SiO_2_/Si substrates.

**Figure 1:**
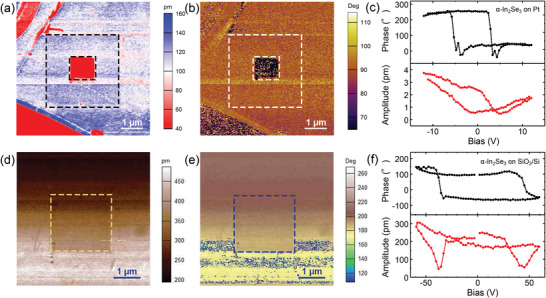
Demonstration of the OOP ferroelectricity of α‐In_2_Se_3_ by PFM. The box‐in‐box images of a) amplitude and b) phase response after the application of an opposite bias: +6 V and −7 V on 3 µm × 3 µm and 1 µm × 1 µm areas, respectively, on a conductive Pt substrate. c) Phase hysteresis loop (black) and amplitude butterfly‐shaped loop (red) under triangular voltage (−12 V to 12 V) applied on ≈25 nm α‐In_2_Se_3_. The box‐in‐box images of d) amplitude and e) phase response after −30 V applied on a 2 µm × 2 µm area of ≈55 nm thick α‐In_2_Se_3_ on a SiO_2_/Si substrate. f) Phase hysteresis loop (black) and amplitude butterfly‐shaped loop (red) under triangular voltage (−60 V to 60 V).

First, +6 and −7 V were applied on a ≈120 nm thick α‐In_2_Se_3_ nanoflake on a Pt substrate (Figure S1a, Supporting Information). The clear box‐in‐box images with distinct color contrast shown in **Figure** **1**a,b indicate that OOP electric dipoles with antiparallel direction were successfully formed between adjacent domains. To exclude the possibility of remnant electrons accumulating on the surface and leading to false ferroelectricity, a triangular sweeping bias was applied on a nanoflake of ≈25 nm thickness. In Figure 1c, off‐field hysteresis loops of phase and amplitude responses, typical characteristics of ferroelectricity, further prove polarization switching. The voltage window obtained from the phase response is 8.16 V, from which, the coercive voltage (*E*_c_) for the polarization to flip can be calculated by *E*_c_ = *V*_c_/d, where *V*_c_ is the absolute difference of the switching voltage between the positive and negative sides, and *d* is the thickness of the α‐In_2_Se_3_ nanoflake. If the air layer is not concerned, the structure would be the simplest circuit with the α‐In_2_Se_3_ nanoflake as the only resistance, and *E*_c_ = 1632 kV cm^−1^.

α‐In_2_Se_3_ Fe‐FETs have been demonstrated on SiO_2_/Si substrates.^[^ ^24^, ^26^, ^27^ ^]^ In the next PFM verification, we chose α‐In_2_Se_3_ directly on SiO_2_/Si substrates. Artificial box images are obtained after applying a −30 V bias on a nanoflake ≈55 nm in thickness (Figure S1b, Supporting Information). Representative hysteresis loops are obtained after sweeping bias through a PFM tip under an electric field. By the same method, in an imaginary circuit consisting of two capacitors: 55 nm α‐In_2_Se_3_ and 50 nm SiO_2_, the voltage shared by α‐In_2_Se_3_ is only 16.4 V and *E*_c_ = 1491 kV cm^−1^, which is of the same order as that obtained from the conducting Pt substrate. Compared to an *E*_c_ value of ≈200 kV cm^−1^ from a previous report,^[^ ^34^ ^]^ this value for both substrates may be due to the presence of air. Regardless, the dependence of the reversible OOP polarization response to an external electric field on the α‐In_2_Se_3_/SiO_2_/Si structure is strong proof of ferroelectricity. Moreover, this lays the fundamentals for understanding the working principle of ferroelectric memory in this work.

**Figure 2:**
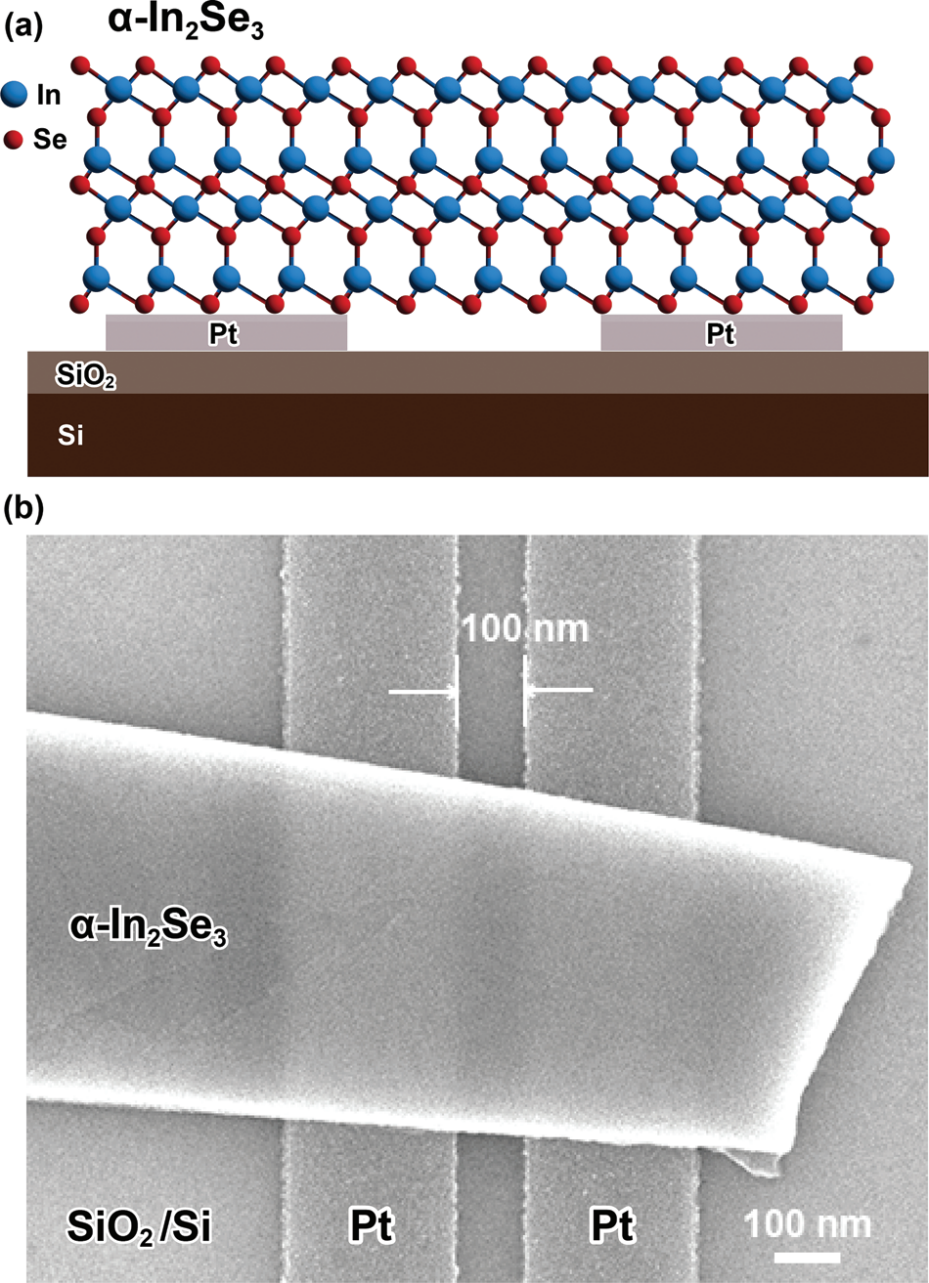
a) Schematic of a Fe‐FET with nanolayered α‐In_2_Se_3_ stamped on top. b) Top‐view FE‐SEM image of lateral Fe‐FET. α‐In_2_Se_3_ nanoflake is stamped on the electrodes. The channel length is 100 nm.

As illustrated in **Figure** **2**a, a 2D lateral Fe‐FET structure was fabricated on a SiO_2_/Si (heavily n‐doped) substrate with Pt nanogap source and drain electrodes by electron beam lithography. The channel material α‐In_2_Se_3_ is mechanically exfoliated from the bulk crystal and then transferred onto the nanogap source and drain electrodes. As shown in Figure 2a, α‐In_2_Se_3_ nanoflake is suspended by source and drain electrodes, creating a vacuum gap between α‐In_2_Se_3_ and SiO_2_ gate dielectric, indicating a non‐contact structure. Field emission scanning electron microscopy (FE‐SEM, Regulus 8230, Hitachi‐High‐Tech) was used to observe the structure. As shown in Figure 2b, the nanoflake is stamped onto the electrodes. After measuring several devices, the average nanogap separation was determined to be 100 nm, which to the best of our knowledge, is the shortest channel length yet reported for an α‐In_2_Se_3_ Fe‐FET.

**Figure 3:**
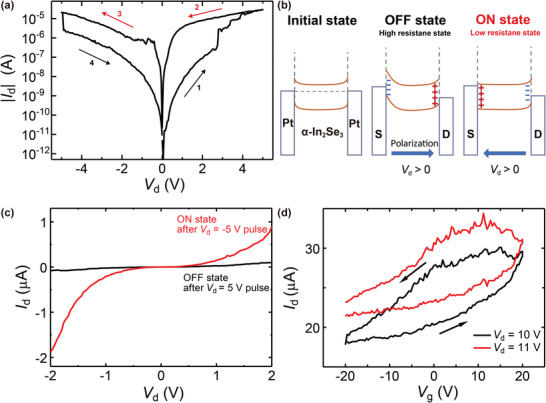
Electrically controlled behaviors of the lateral α‐In_2_Se_3_ Fe‐FET. a) Hysteresis loop of *I*_d_–*V*_d_ curve of the planar Fe‐FET with 29 nm thick α‐In_2_Se_3_ (Figure S2, Supporting Information). b) Band diagram of the ferroelectric switching mechanism. c) Clear on/off states after opposite pulses of ±5 V for 1 s. d) *I*_d_–*V*_g_ curves show hysteresis at *V*_d_ = 10 V (black) and 11 V (red). All measurements are in a dark and vacuum environment.

The double‐sweep *I*_d_–*V*_d_ hysteresis loops with a ±5 V bias in a dark and a vacuum environment is shown in **Figure** **3**a. In these measurements, a hysteresis loop was achieved via *V*_d_ sweeping in the sequence from Steps 1 to 4 without back gate (Si) voltage (*V*_g_). Notably, when *V*_d_ increased from 50 to 750 mV, the on/off ratio is over 10^3^; at *V*_d_ = 50 mV, the on/off ratio is more than 9000.

The memory effect relies on *V*_d_ changing the channel conductance of α‐In_2_Se_3_. IP polarization is the origin of this memory effect for the following explanations. This result is obtained on a two‐terminal device with vdW nanolayered α‐In_2_Se_3_, where the lateral electric field is applied from the nanogap electrodes to the bottom‐contact α‐In_2_Se_3_ in the absence of a gate voltage. Because there are no electrodes on top of α‐In_2_Se_3_, it is hard to generate OOP polarization inside the materials at the beginning. To the best of our knowledge, this is the first demonstration of the ferroelectric memory effect originating from the IP polarization of α‐In_2_Se_3_ to achieve an on/off ratio of over 10^3^. Roughly comparing with the *I*_d_–*V*_d_ result of α‐In_2_Se_3_ ferroelectric memory with 1 µm in channel length (Figure S3, Supporting Information), 100 nm channel length can lower the write voltage, meanwhile, increase the on/off ratio through a faster and more complete polarization switching via a narrower channel length.

The memory mechanism can be explained as IP ferroelectricity that triggers modulation of the Schottky barrier height at the interfaces between electrodes and the channel material α‐In_2_Se_3_ for the reason that in our proposed non‐contact bottom contact structure, α‐In_2_Se_3_ nanoflake is contacting nanogap source and drain, but suspended over a vacuum gap upon SiO_2_ gate dielectric. The energy band diagrams of the initial‐, off‐, and on‐states are shown in Figure 3b. When the drain voltage is swept from negative to positive below the coercive voltage (Steps 4 to 1 in Figure 3a), the IP polarization inside α‐In_2_Se_3_ is maintained, leading to the accumulation of negative and positive charges at the source and drain/material interfaces, respectively. The Schottky barrier height at the source/material interface is then lifted and the resistance is high, which is the off state. When the drain voltage becomes larger than the coercive voltage, the direction of IP polarization is reversed, which lowers the Schottky barrier height at the source/material interface due to the accumulation of positive charges, which is the on state with low resistance (Steps 2 to 3 in Figure 3a). This IP polarization is maintained until the negative drain voltage becomes larger than the applied coercive voltage.

The asymmetric *I*_d_–*V*_d_ hysteresis loops in Figure 3a for our symmetric systems consisting of identical Pt electrodes can be explained as follows. There are two interfacial layers between a nanoflake and a pair of Pt nanogap electrodes by stamp, which causes different interfaces in effective thickness and/or effective dielectric constant.^[^ ^35^ ^]^ The area of the bottom‐contact α‐In_2_Se_3_ on each electrode is also different and could lead to an unbalanced positive and negative current density at the interfaces. Furthermore, concerning the n‐type semiconducting property of α‐In_2_Se_3_, the asymmetric *I–V* curve is likely driven by the reversible accumulation or depletion of carriers in α‐In_2_Se_3_ at the two interfaces, which has an impact on the Schottky barrier height and polarization‐induced conductivity.^[^ ^36^ ^]^ On the positive side, the abrupt change of current at *V*_d_ = 2.8 V is smaller than that at *V*_d_ = −4.9 V on the negative side, which suggests the facilitation of electrons in α‐In_2_Se_3_. Lastly, it is presumable that the slight shift or offset of the *I–V* hysteresis loops is attributable to the original polar defects of α‐In_2_Se_3_^[^ ^37^, ^38^ ^]^ and the OOP polarization that promotes the asymmetrical distribution of the interface electrons.^[^ ^39^ ^]^

Upon the application of an external DC electric field over the coercive electric field of α‐In_2_Se_3_, the polarity can be controlled, allowing randomly oriented polarizations to align with the direction of the electric field, and thereby, manually switching between the desired on or off state. To further investigate the on/off states, we selected the negative voltage as the on state. In a linear scale (Figure 3c), −5 V is applied as a set pulse and 5 V as a reset pulse for 1 s. A sweeping voltage from −2 to 2 V is applied to read. On/off currents are established repeatedly with almost no fluctuation after four continuous cycles from −2 to 2 V, where the unchanged on/off states are largely depending on the history bias that initially induces left or right directional polarization. These results all identified the potential for our device to realize the IP memory effect using only two terminals, source, and drain.

Additionally, a back gate can induce a drain current. *I*_d_–*V*_g_ hysteresis loops demonstrate the feasibility of a Fe‐FET. Figure 3d shows the n‐type anticlockwise memory window under a *V*_g_ sweep from −20 to 20 V while *V*_d_ is fixed. The vertical electric field emanating from the back gate of the Si substrate initiates OOP polarization, which induces further IP polarization. It is expected that intercorrelation occurs between IP and OOP polarization. The distorted *I*_d_–*V*_g_ curves can be explained that our designed structure, as mentioned in Figure 2a, has a vacuum gap between α‐In_2_Se_3_ and the bottom SiO_2_ gate dielectric, resulting in a weak coupling between the bottom gate and the ferroelectric channel. This result in advance consolidates that IP polarization plays a main role in controlling ferroelectricity memory in our proposed devices.

**Figure 4:**
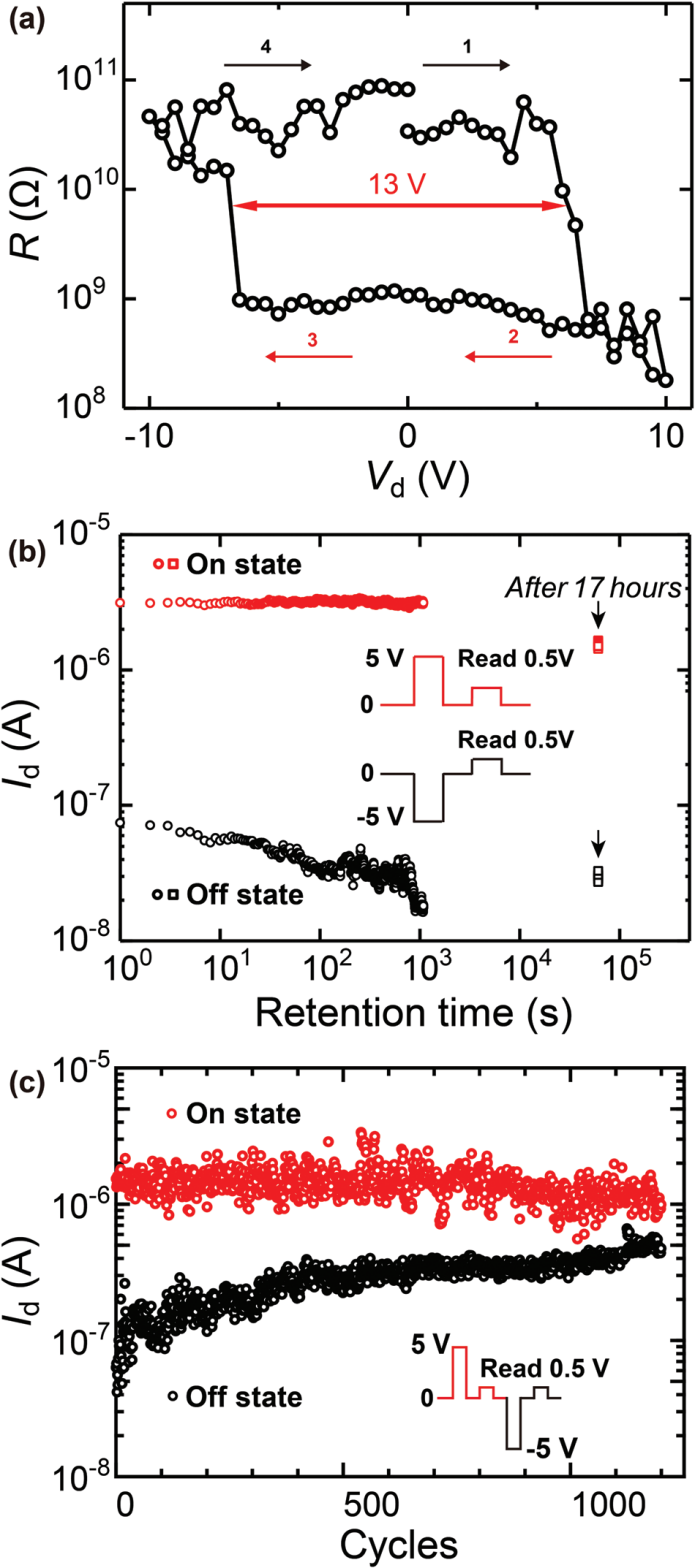
Memory performance of the planar α‐In_2_Se_3_ Fe‐FET. The thickness of α‐In_2_Se_3_: 47 nm (Figure S4, Supporting Information). a) Resistance (*R*) under read voltage of 0.5 V as a function of write voltage (*V*_d_) with a memory window of 13 V. b) Retention: stable on and off states over one order of magnitude after 17 h. The write and read pulse width is 1 s. c) Endurance: on and off states over 1200 cycles. The write and read pulse width is 200 ms. The set and reset voltages are 5 V and −5 V, respectively, and the read voltage is 0.5 V in both retention and endurance tests.

Our device comprising 47 nm α‐In_2_Se_3_ fulfills the needs of memory operation under various pulse sequences. First, sufficient write pulses from the drain tune the IP polarization with an amplitude ranging from −10 to 10 V. After every write pulse, a fixed read pulse at 0.5 V is applied. Consequently, the *R*–*V*_d_ curve shown in **Figure** **4**a exhibits a hysteresis loop with an on/off ratio of 40 and a memory window of 13 V. This 13 V memory window of IP polarization corresponds to *E*_c_ = 650 kV cm^−1^, which is smaller than *E*_c_ of OOP polarization measured by PFM in Figures 1C. This tendency is consistent with a previous report in Li's work.^[^ ^40^ ^]^ It notes that bottom contact IP polarization Fe‐memory with a big 13 V memory window has the potential for expanding the system's memory capacity, reducing power consumption, and improving reliability.

Retention evaluated after 17 h is shown in Figure 4b. The write and erase voltages were 5 and −5 V for 1 s, respectively, to initial the on/off states. Next, 0.5 V *V*_read_ for 1 s is applied for 1000 cycles. After 17 h, another 10 pulses of read voltage are applied. Two stable states of read current are built with almost no decreases, which reveals the non‐volatile characteristics of our IP ferroelectric memory. Figure 4c shows the endurance performance under 200 ms programmed pulse sequences of write (5 V), read (0.5 V), erase (−5 V), and read for 1200 cycles. Endurance over 10^3^ cycles with separate on/off states is solid evidence for the lateral memory effect of our bottom contact device.

## 3 Conclusion

Here, the IP ferroelectric memory effect of 100 nm channel‐length 2D vdW ferroelectric semiconductor α‐In_2_Se_3_ was demonstrated. By introducing bottom‐contact type ferroelectric memory structure, resistive switching, and non‐volatile functionality due to IP polarization inside α‐In_2_Se_3_ were rationalized as the modulation of Schottky barrier height between the interfaces of channel materials and electrodes. On/off ratio of 10^3^, a large memory window of 13 V at *V*_d_ between ±6.5 V, retention lasting 17 h, and endurance over 10^3^ cycles were achieved. With the gate bias, the device exhibited a hysteresis loop because of the intercorrelation between IP and OOP polarization. The ferroelectricity of α‐In_2_Se_3_ on Si/SiO_2_ was also demonstrated through PFM observations. Overall, these results strengthen the concept of IP ferroelectric non‐volatile memory. With a bottom contact structure of 100 nm in channel length, massive integration becomes promising considering the simplified construction of next‐generation electronics.

## 4 Experimental Section

### Device Fabrication

Bottom Ti/Pt electrodes were fabricated by a lift‐off process combining electron‐beam lithography (EBL) and electron beam (EB) evaporation.^[^ ^32^ ^]^ An EB resist (ZEP520A, Zeon) diluted with anisole (ZEP‐A, Zeon) was coated onto cleaned Si(100) (525 µm)/SiO_2_ (50 nm) substrates using a spin coater. The devices were pre‐baked on a hot plate. An EBL apparatus (ELS‐7500EX, Elionix) was used to pattern the nanogap structure with 100 separations on the resist‐coated substrates. After the development of the resist, Ti/Pt 3/10 nm thick was deposited by EB evaporation. A standard photolithographic process using an MA‐20 mask aligner (MIKASA) was carried out to fabricate electrode probers. S1818 resist was coated onto substrates followed by the deposition of 5 nm Ti and 40 nm Pt through EB evaporation. Acetone was used to lift off S1818 resist, and Ti/Pt nanogap electrodes with both leads and pads were obtained. After the lift‐off process, the substrate was cleaned via a 20‐min UV ozone treatment to remove the resist residue and ensure good contact with multilayer α‐In_2_Se_3_ nanoflakes, which were mechanically exfoliated from bulk single crystals purchased from HQ Graphene, Inc., and then transferred by polydimethylsiloxane onto the bottom electrodes. The thickness of the α‐In_2_Se_3_ nanoflakes was decreased using Scotch Tape (3M) company.

### Materials Characterization

PFM measurements were conducted using a commercial atomic force microscope (Asylum Research MFP‐3D) with Pt/Ir‐coated Si cantilever tips (NanoAndMore USA, nominal spring constant 2.8 N m^−1^) to measure the ferroelectricity of α‐In_2_Se_3_ nanoflakes exfoliated onto both conductive Pt film deposited on SiO_2_/Si substrate and bare SiO_2_/Si substrate. Box‐in‐box measurements were conducted by applying opposite directional voltages on chosen outer to inner squares. The OOP PFM signal was recorded using a driving frequency of 280 kHz and a drive amplitude of 3000 mV. A bidirectional bias sweep between −12 V and 12 V (Pt substrate) and −60 V and 60 V (Si/SiO_2_ substrate) was applied to measure the hysteresis loops in dual‐a.c.‐resonance‐tracking (DART)‐PFM.

### Electrical Measurements

The electrical property curves of planar bottom contact devices were obtained using a semiconductor parameter analyzer (B1500, Keysight) connected to a mechanical helium refrigerator‐type prober station (GRAIL 10‐LOGOS01S, Nagase) at room temperature under vacuum (≈10^−4^ Pa) in a dark environment.

## Conflict of Interest

The authors declare no conflict of interest.

## Supporting information

Supporting Information

## Data Availability

The data that support the findings of this study are available from the corresponding author upon reasonable request.
