# Supplementary material for: Bottom Contact 100 nm Channel‐Length α‐In2Se3 In‐Plane Ferroelectric Memory
Source: Adv Sci (Weinh). 2023 Aug 11;10(29):2303032. doi: 10.1002/advs.202303032 (PMC10582452; doi:10.1002/advs.202303032)
Supplement: Supplementary file 1 — Supporting Information [file ADVS-10-2303032-s001.pdf]

ADVANCED SCIENCE
Open Access

# Supporting Information

for *Adv. Sci.*, DOI 10.1002/advs.202303032

Bottom Contact 100 nm Channel-Length $\alpha$-$In_2Se_3$ In-Plane Ferroelectric Memory

*Shurong Miao, Ryosuke Nitta, Seiichiro Izawa and Yutaka Majima**

# Supporting Information

**Bottom Contact 100 nm Channel-Length α-$In_2Se_3$ In-Plane Ferroelectric Memory**

*Shurong Miao, Ryosuke Nitta, Seiichiro Izawa, and Yutaka Majima*[*]

S. Miao, R. Nitta, S. Izawa, Y. Majima
Laboratory for Materials and Structures, Institute of Innovative Research,
Tokyo Institute of Technology, Yokohama, Kanagawa 226-8503, Japan
E-mail: majima@msl.titech.ac.jp

S. Izawa
Joining and Welding Research Institute, Osaka University, Ibaraki, Osaka 567-0047, Japan

Supporting Information 1.

PFM topography images.

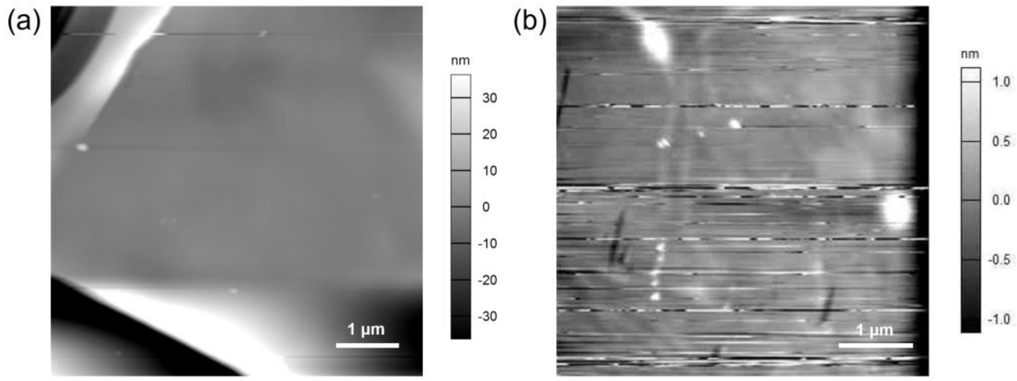


Figure S1. The topography of α-$In_2Se_3$ nanoflake observed by PFM (a) on Pt substrate, thickness of α-$In_2Se_3$ nanoflake: ~120 nm, and (b) on $SiO_2$/Si substrate, thickness of α-$In_2Se_3$ nanoflake: ~55 nm.

Supporting Information 2.

The actual α-$In_2Se_3$ in-plane device in Figure 3 observed by atomic force microscope (AFM) and field emission scanning electron microscope (FE-SEM).

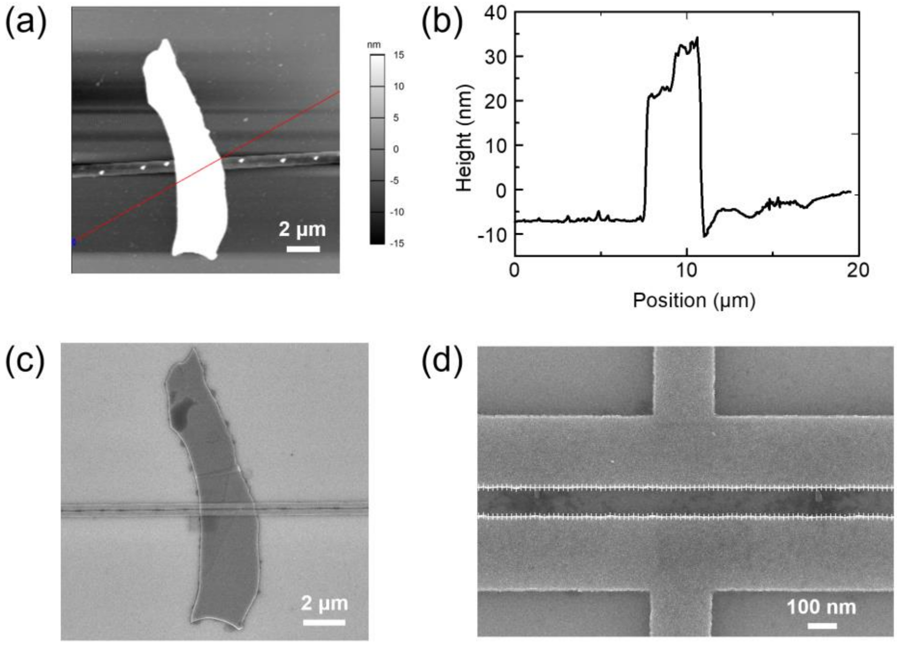


Figure S2. (a) The topography of α-$In_2Se_3$ nanoflake observed by AFM. (b) The thickness of this nanoflake about 29 nm. (c) The device observed by FE-SEM and (d) The channel length measured by FE-SEM.

Supporting Information 3.

α-$In_2Se_3$ in-plane device with 1 μm in channel length.

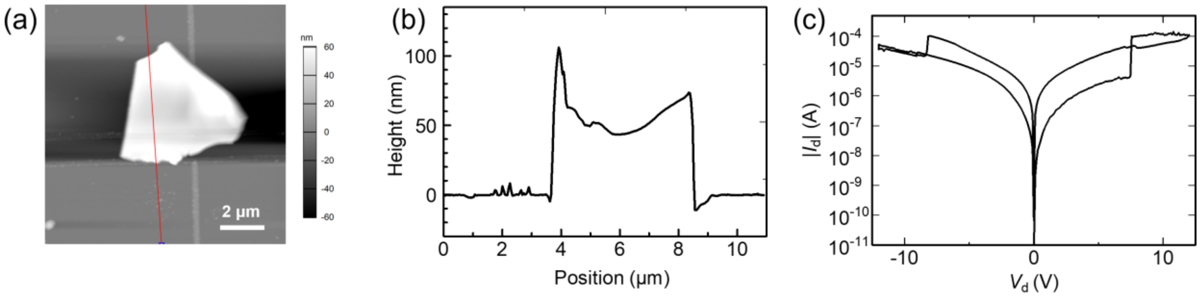


Figure S3. (a) The topography of 1 μm α-$In_2Se_3$ in-plane device observed by AFM. (b) The thickness of this nanoflake in the nanogap is about 52 nm. (c) $I_d$-$V_d$ curve of this 1 μm α-$In_2Se_3$ in-plane device.

Supporting Information 4.

The actual α-$In_2Se_3$ in-plane device in Figure 4 observed by AFM and FE-SEM.

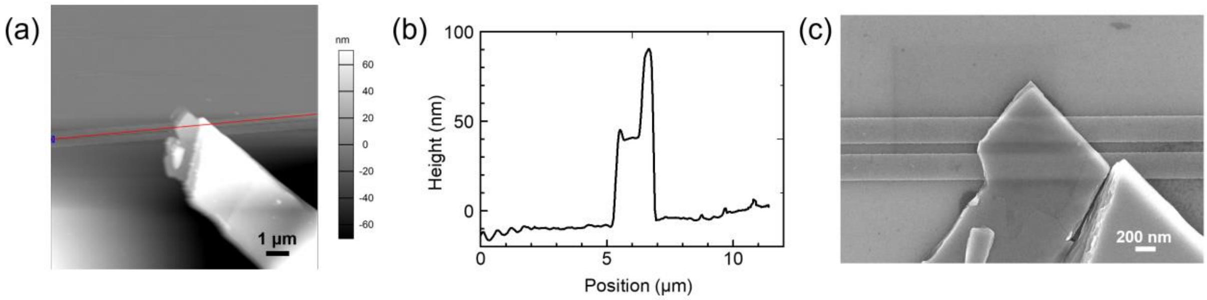


Figure S4. (a) The topography of α-$In_2Se_3$ nanoflake observed by AFM and (b) The thickness of this nanoflake in the nanogap is about 47 nm. (c) The device observed by FE-SEM.
